# Physical Activity Levels of Community-Dwelling Older Adults During Daily Life Activities: A Descriptive Study

**DOI:** 10.3390/healthcare12242575

**Published:** 2024-12-21

**Authors:** Dieuwke van Dartel, Ying Wang, Johannes H. Hegeman, Miriam M. R. Vollenbroek-Hutten

**Affiliations:** 1 Biomedical Signals and Systems Group, University of Twente, 7522 NB Enschede, The Netherlands; 2 Department of Trauma Surgery, Ziekenhuisgroep Twente, 7609 PP Almelo, The Netherlands; 3 Board of Directors, Medisch Spectrum Twente, 7512 KZ Enschede, The Netherlands

**Keywords:** hip fracture, older adults, physical functioning, physical activity, continuous monitoring, wearable devices

## Abstract

Background/Objectives: Measuring the physical functioning of older hip fracture patients using wearables is desirable, with physical activity monitoring offering a promising approach. However, it is first important to assess physical activity in healthy older adults. This study quantifies physical functioning with physical activity parameters and assesses those parameters in community-dwelling older adults. The results are compared with the results from one case participant 2 months post-hip fracture surgery. Methods: Twenty-four community-dwelling older adults (aged ≥ 80) participated. The acts of moving around the house, toileting, getting in/out of bed, and preparing meals was quantified by total time, time spent sitting, standing, and walking, number of transfers, and intensity of physical activity. MOX and APDM sensors measured the intensity of physical activity, with the tasks performed in a living lab while video-recorded. The case participant’s total time and intensity of physical activity were measured for walking to a door and getting in/out of bed. Results: Preparing meals showed the longest total time and time spent standing/walking, while moving around the house and getting in/out of bed had the highest intensity of physical activity. Only getting in/out of bed required sitting. The physical activity parameters varied among participants, with very active participants completing tasks faster. The case participant had longer total times and lower intensities of physical activity two months post-surgery compared to before the fracture. Conclusions: This study provides initial insights into the physical activity levels of community-dwelling older adults. It represents the beginning of more efficient and continuous monitoring of physical functioning.

## 1. Introduction

In recent years, wearable devices have become more common in monitoring older patients during hip fracture rehabilitation [1,2,3,4,5,6,7,8,9,10,11,12,13,14,15,16]. Multiple studies were performed describing the level of physical activity of older hip fracture patients [7,8,9,10,11,12,13,14,15,16] or describing the relevance of physical activity in the recovery process [2,3,4,5,6,7]. From those studies, it is known that the level of physical activity in the first three months after hip fracture surgery is limited [13,14,15,16,17]. Furthermore, it is known that more physical activity during rehabilitation results in a faster recovery in physical functioning [4,5,6]. Physical functioning includes mobility and Activities of Daily Living (ADL), in which mobility is described as the ability to move and is measured by walking ability, muscle strength, and balance. The ultimate goal of hip fracture treatment is to recover patients in such a way that they regain their prefracture level of physical functioning. Clinimetric tests, such as the Functional Independence Measure (FIM) [18,19,20,21,22,23,24,25], Barthel Index (BI) [24,25,26,27,28,29,30,31,32], or the Timed Up and Go test (TUG) [8,14,23,24,25,33,34,35,36], are currently the gold standard for monitoring recovery in physical functioning. However, monitoring the recovery in physical functioning using wearable devices is considered more desirable since current clinimetric tests have multiple limitations. Clinimetric tests are subjective, static measurements and often just represent a point in time. Additionally, patients may lack the mobility to perform clinimetric tests, leading to missing data. Even when patients are capable of completing a test, clinimetric tests can be time-consuming, thereby increasing the workload on healthcare professionals. Using wearable devices can provide continuous 24/7 monitoring of patients, which enables healthcare professionals to track patients’ progress in a fast and objective way. However, unlike physical activity, there are still no studies monitoring physical functioning in older patients after hip fracture surgery using wearable devices.

Monitoring physical functioning using wearable devices is considered complex. The direct monitoring of physical functioning requires activity recognition algorithms based on continuous parameters derived from multiple wearable devices placed at different locations on the human body [37,38,39,40,41]. This is not considered feasible in an older population since using multiple wearables corresponds with a high burden on the older population, which is not considered viable. Therefore, an alternative method to monitor physical function using wearable devices must be sought. Studies in other clinical fields than hip fracture rehabilitation have shown that monitoring physical functioning with wearable devices can also be achieved by quantifying it with physical activity parameters [40,42]. Physical activity underlies most tasks related to physical functioning and is, therefore, considered a potential monitoring method. In a study by Bidargaddi and Sarela, for example, physical activity parameters were derived from accelerometer data to assess the progress of patients during cardiac rehabilitation. The monitoring of mobility was quantified by monitoring the time spent walking, walking speed, frequency of walking, and walking control [40]. Sun et al. used accelerometer data to quantify functional recovery in patients with abdominal cancer by monitoring the number of steps per minute [42]. Both studies showed valid ways to continuously monitor physical functioning by physical activity parameters, indicating that this way of monitoring also seems to be a suitable method for assessing physical functioning in an older population after a hip fracture.

However, currently, only basic ADL tasks, such as walking, sitting, or making transfers, have been quantified with physical activity parameters. More complex tasks important in the recovery process of older hip fracture patients, such as getting in and out of bed, are not quantified yet, and for this, more research is recommended. In addition, to gain insight into the level of functional recovery of hip fracture patients through physical activity monitoring, it is important to first understand the physical activity levels of healthy comparable individuals during mobility and ADL tasks. In this way, a reference point can be established that can be subsequently used to assess the level of recovery in hip fracture patients. Therefore, this study aims to quantify physical functioning with physical activity parameters and to assess those parameters in community-dwelling older adults aged 80 years or older through an experiment conducted in a real-life simulated environment. Additionally, a case study will be described in which the physical activity parameters obtained from the community-dwelling older adults will be compared with the physical activity parameters of a single participant who initially participated in this study and was subsequently admitted to our hospital with a hip fracture. This unique case study is the first assessment of the level of recovery two months after hip fracture surgery based on the measured physical activity parameters.

## 2. Materials and Methods

### 2.1. Study Design and Participants

**Figure 1:**
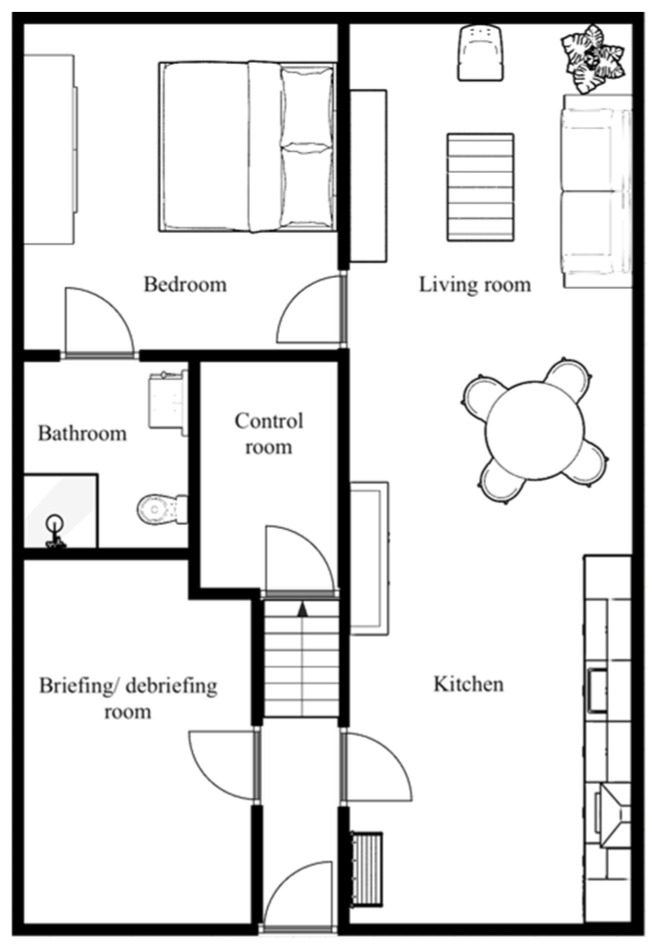
Floor map of the eHealth House. More details can be found on the following website: https://www.utwente.nl/en/techmed/facilities/htwb-labs/ehealth-house/# (accessed on 2 December 2022).

This study was a prospective observational experimental study performed from May 2021 to November 2021 at the University of Twente, Enschede, The Netherlands. The study was executed at the eHealth House of the TechMed Simulation Centre, which offers a living lab (furnished apartment) with the ability to perform experiments in a real-life simulated environment (Figure 1). A total of 24 participants aged 80 or older, who were physically able to perform the study were enrolled. Participants who were mentally not able to sign the informed consent form, who had mobility disorders so severe that they were unable to walk, or participants who did not speak the Dutch language were excluded. All participants were asked to visit the eHealth House once to perform the study. The study was approved by the Medical Research Ethics Committee (MREC) Arnhem–Nijmegen and the ethical committee Natural Sciences and Engineering Sciences of the University of Twente. The study was considered as not subject to the Medical Research Involving Human Subjects Act (WMO). All participants gave written informed consent to participate.

### 2.2. Physical Activity Parameters

Mobility and ADL tasks, which reflect physical functioning and are relevant to the recovery process of older patients after hip fracture surgery, were performed in this study. Those tasks were moving around the house, toileting, getting in and out of bed, and preparing meals. These tasks were considered important since hip fracture patients need to regain independence in performing those tasks in order to return to their prefracture living situation. To quantify these tasks, the tasks were first split into smaller activities that are needed to perform the tasks (Table 1). For instance, for toileting, the following activities were considered needed: walk to the toilet, stand still in front of the toilet, drop pants, sit down on the toilet, stand up from the toilet, put pants back on, and walk back from the toilet. Based on those smaller activities, physical activity parameters were identified that underlie the smaller activities. Those physical activity parameters are total time to perform the task, time spent sitting, time spent standing, time spent walking, number of transfers, and intensity of physical activity.

### 2.3. Wearable Devices

**Figure 2:**
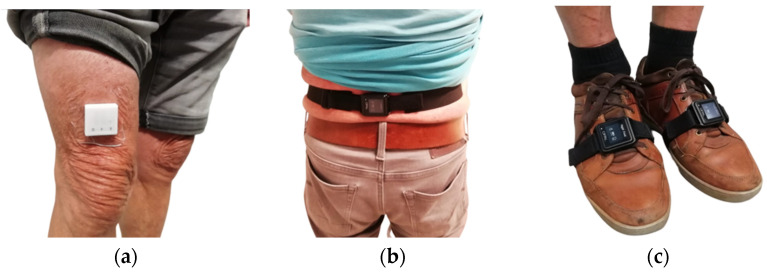
Wearable devices used in this study: (**a**) shows the MOX on the anterior thigh; (**b**) shows the APDM Opal on the lower back; (**c**) shows the ADPM Opals on both feet.

Two wearable devices were used in this study to continuously monitor the physical activity of the enrolled participants: the MOX activity monitor (Maastricht Instruments BV, Maastricht, The Netherlands) and the APDM wearable sensor (Hankamp Rehab BV, Enschede, The Netherlands). The MOX activity monitor (model MMOXX) is a small, single-unit waterproof device that contains a tri-axial accelerometer to monitor a participant’s physical activity continuously. Physical activity parameters that can be measured by the MOX are the intensity of physical activity and sedentary (lying, sitting), standing, and dynamic activities. Raw acceleration data were measured with a sample frequency of 25 Hz. During this study, the MOX was attached to the anterior thigh using an adhesive plaster (Figure 2a) [43]. With the use of IDEEQ software provided by Maastricht Instrument BV, raw acceleration data were collected for further data analysis.

The APDM sensor consists of multiple small sensors, Opals, which can be placed at different locations on the body to assess, for example, the number of steps, gait speed, gait cycle duration, stride length, step duration, and balance (lumbar range of motion, postural sway). Opals are small, single-unit devices that contain a tri-axial accelerometer, a tri-axial magnetometer, and a tri-axial gyroscope. Raw data were measured with a sample frequency of 128 Hz. For lower limb gait and balance analysis, three Opals were recommended by Hankamp Rehab BV: one lumbar (Figure 2b) and two on the foot (Figure 2c) [44]. These were used in this study. With the use of Mobility Lab software provided by APDM, raw data were collected for further data analysis. https://www.accelerometry.eu/solutions/wearable-data-processing-analysis/ideeq-data-visualization/ (accessed on 3 November 2024).

### 2.4. Study Procedure

The study consisted of two parts. During the first part, the wearable devices were placed on the participant’s body; the MOX at the anterior thigh, 10 cm above the right knee (Figure 2a), and the APDM Opals on both feet and the lower back (Figure 2b,c). Furthermore, some general questions were asked about the participant’s characteristics, like age, gender and daily physical activity levels. The daily physical activity levels were classified as a sedentary, slightly active, moderately active, or very active lifestyle [45]. A sedentary lifestyle primarily consisted of only performing ADL tasks; a slightly active lifestyle consisted of performing ADL tasks, household chores, and walking; a moderately active lifestyle consisted of performing ADL tasks, household chores, walking, cycling, and fitness lessons, especially for older adults; and a very active lifestyle consisted of performing ADL tasks, household chores, walking, cycling, and exercising multiple times per week. The researchers extensively asked about the various aspects of daily physical activity in order to provide a detailed understanding of the daily physical activity levels and minimize the risk of inaccuracies, including the type of sport the participant engages in, the frequency, which other physical activities participants perform, and whether they carry out household chores.

The second part of the study focused on performing the mobility and ADL tasks. The activities shown in Table 1 describe each task in more detail. Moving around the house was divided into three tasks: (1) a walk to the front door, (2) a walk to the bedroom, and (3) a walk to the kitchen. Preparing meals was divided into two tasks: (1) preparing a small meal and (2) grabbing something to drink. Participants were allowed to perform all seven tasks in their own order and at their own pace. Participants wore the wearable devices continuously during the entire experiment. Furthermore, video recordings were used as a gold standard to annotate the observed physical activities during the experiment.

### 2.5. Data Analyses

For all participants, the following physical activity parameters were calculated for each mobility and ADL task: total time, time spent sitting, time spent standing, time spent walking, number of transfers, and the intensity of physical activity. The total time, time spent sitting, time spent standing, time spent walking, and the number of transfers were calculated based on the video recordings. For all participants, the video recordings during the measurement were annotated by two independent researchers, who were both trained in how to annotate the video recordings. For every second of the measurement, it was annotated whether the participant was sitting, standing, walking, or making a transfer. Furthermore, the start and end times of all performed tasks were annotated. Discrepancies between the two independent researchers were checked by a third independent researcher to make a final decision. Then, the total time for each task was calculated by taking the difference between the end time and the start time of the task. The time spent sitting, the time spent standing, and the time spent walking were each calculated by counting the seconds of sitting, standing, and walking for each task based on the annotations. The number of transfers was calculated by counting how many transfers were performed during a task. Transfers included any activity in which a participant went from sit to stand, stand to sit, stand to lie, lie to stand, sit to lie, or lie to sit. When participants were able to get in or out of bed in one smooth movement, one transfer was counted (stand to lie or lie to stand). When participants needed to sit first before they lay down or before they got up, we counted two transfers: stand to sit and sit to lie or vice versa.

The intensity of physical activity was calculated for each participant based on the accelerometer data from the wearable devices. Since the calculation of the intensity of physical activity can be affected by the sensor location on the body, it was chosen to calculate this parameter for both the MOX at the anterior thigh, as well as the APDM at the lower back, and the APDM at the right foot. To calculate the intensity of physical activity, raw acceleration data were first pre-processed. For the MOX, raw acceleration data were pre-processed by a moving average filter with a window length of 0.12 s and a fourth-order Butterworth High Pass filter with a cut-off frequency of 1 Hz [46]. For the APDM, raw acceleration data were pre-processed by a fourth-order Butterworth Bandpass filter between 0.25 Hz and 20 Hz [47,48]. The intensity of physical activity was calculated for every second of the measurement by determining the signal magnitude area (SMA) of the acceleration data. The mean of the intensity of physical activity was then calculated for each task and expressed in counts per second (cps) [46]. All analyses were performed using Matlab (R2017b, MathWorks Inc., Natick, MA, USA).

### 2.6. Descriptive Analyses

Patient characteristics, like age, gender, and daily physical activity levels, were described using descriptive statistics. Continuous variables were described as mean with the standard deviation (SD) or as median with the interquartile range (IQR) in the case of non-parametric data. Categorical variables were shown as numbers with the corresponding percentages. The physical activity parameters were also described using descriptive statistics. For all tasks, the physical activity parameters were described for the total number of participants in order to establish a reference point. Additionally, two groups were created based on the daily physical activity levels. The first group consisted of participants with a very active lifestyle, while the second group consisted of participants with a moderately active, slightly active, or sedentary lifestyle and was defined as the sedentary to moderately active group. The total time and number of transfers were described for both groups in order to assess whether daily physical activity levels affected the physical activity parameters. In the case of parametric data, the physical activity parameters were described as mean with SD. In the case of non-parametric data, the parameters were described as median with IQR. The minimum and maximum for all physical activity parameters were also described for each task.

### 2.7. Case Study

During the course of this study, one of the participants, who had already finished the measurements of this study, fell at home and was admitted to our hospital with a hip fracture. After hip fracture surgery, this participant participated in one of our other research projects called the “Up&Go after a hip fracture” project, which focused on the continuous monitoring of physical activity in older patients after hip fracture surgery during rehabilitation at a skilled nursing home [3,4]. For this project, the participant’s physical activity was continuously monitored during the complete rehabilitation at the skilled nursing home using the MOX activity monitor. The intensity of physical activity was calculated to obtain insight into the participant’s recovery. The Up&Go project was approved by the MREC Twente and by the institutional review board of Ziekenhuisgroep Twente (ZGT), The Netherlands. The MREC Twente considered the project as not subject to the WMO. The participant gave written informed consent to participate in the Up&Go project. Furthermore, the participant gave written permission to use the data from the Up&Go project for other research purposes. This gave us the opportunity to compare the participant’s intensity of physical activity at discharge from rehabilitation with the participant’s intensity of physical activity prior to the hip fracture in order to do a first assessment of the level of recovery.

To compare the levels of physical activity, annotations of mobility and ADL tasks were also made during rehabilitation after hip fracture surgery. On the day before discharge, we noted the moment when the participant got in and out of bed and when the participant walked to the door of the room, which was approximately half of the distance to the front door of the eHealth House. From the MOX data, the total time and intensity of physical activity were calculated. The total time and intensity of physical activity were compared with the data obtained during the measurements at the eHealth House. Furthermore, the total time and intensity of physical activity from the single participant at the moment of discharge from rehabilitation were also compared with the total time and intensity of physical activity of the total group of community-dwelling older participants.

## 3. Results

### 3.1. Participants

A total of 24 participants were enrolled in this study. The median (IQR) age of those participants was 82 (81–85) years old, and 13 participants (54.2%) were female. The daily life physical activity levels varied amongst the enrolled participants, with 8 participants having a very active lifestyle, 12 participants having a moderately active lifestyle, 3 participants having a slightly active lifestyle, and 1 participant having a sedentary lifestyle. A total of five participants had missing data on one or more mobility and ADL tasks due to two reasons: performing two tasks at the same time or finishing one task and immediately continuing to the next task without sitting in between.

### 3.2. Physical Activity Parameters

**Figure 3:**
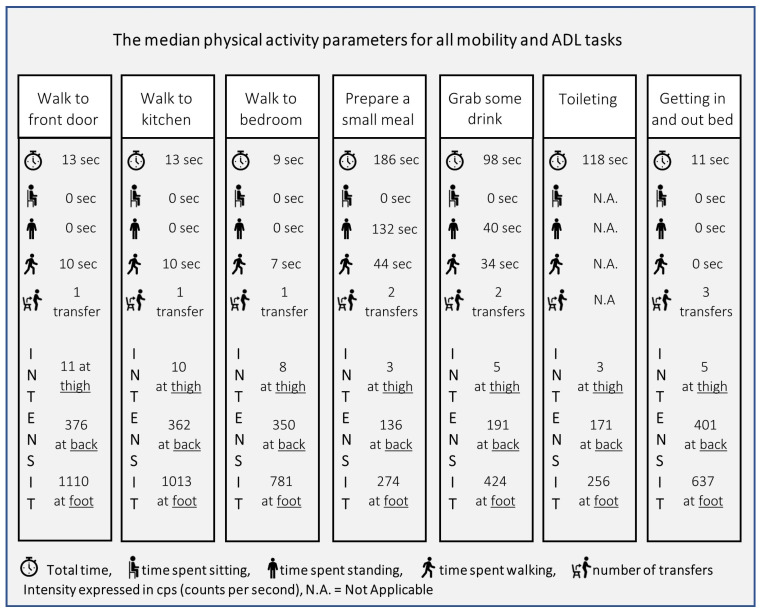
The median physical activity parameters for each task performed during this study.

The median values of all physical activity parameters for all seven mobility and ADL tasks are shown in Figure 3, and more detailed information is shown in Table 2. Results show that the total time, time spent standing and time spent walking were the longest for preparing a meal. Results also show that participants were able to perform most tasks without a period of rest in between, since the time spent sitting was zero seconds for most tasks. Only for getting in and out of bed, the time spent sitting varied between a minimum of zero seconds and a maximum of 14 s (Table 2), also resulting in the number of transfers varying between 2 and 4 transfers. The intensity of physical activity measured at the thigh and right foot was highest during moving around the house. When measured at the lower back, the intensity of physical activity was highest for getting in and out of bed. Table 2 also shows the variability in the physical activity parameters between the participants. Varying results are shown amongst the participants for the total time, time spent standing, and intensity of physical activity during preparing a meal and toileting. Participants also showed varying results in the total time of getting in and out of bed.

Table 3 shows the total time and the number of transfers for the participants in the group with a very active lifestyle and for the participants in the sedentary to moderately active group. Results show that participants with a very active lifestyle have the shortest total time for all tasks. Furthermore, for getting in and out of bed results show that participants with a very active lifestyle were able to do this task with only two transfers, whereas the other participants needed three transfers, indicating that they needed a period of sitting in between.

### 3.3. Case Study

**Figure 4:**
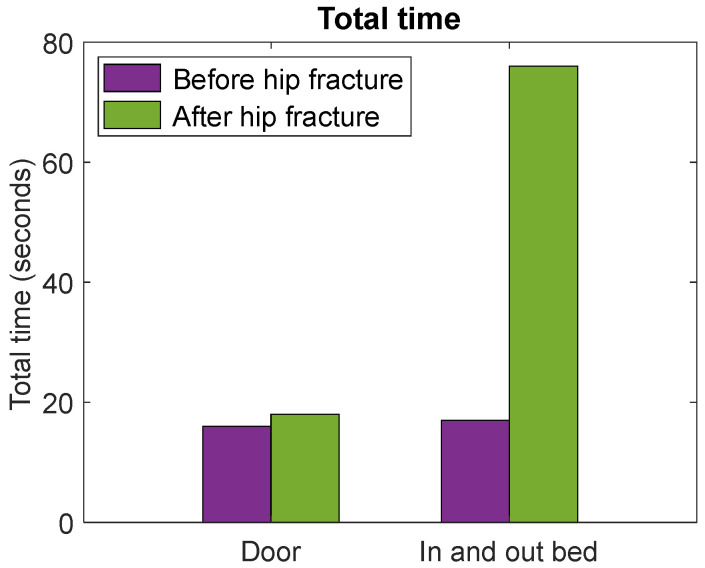
The total time of walking to the door (**left**) and getting in and out of bed (**right**) was measured in the case participant during the measurements at the eHealth House before the hip fracture and at the moment of discharge after the hip fracture. The distance to the door at the rehabilitation department was approximately half the distance to the door at the eHealth House.

**Figure 5:**
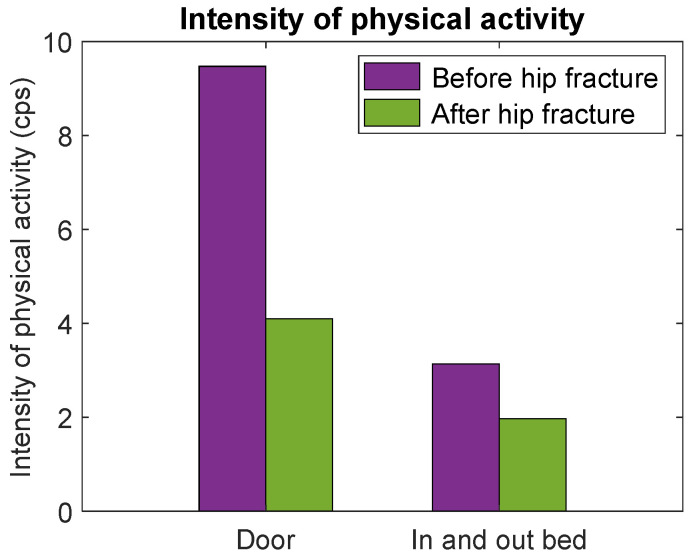
The intensity of physical activity at the thigh during walking to the door (**left**) and going in and out of bed (**right**) was measured in the case participant during the measurements in the eHealth House before the hip fracture and at the moment of discharge after the hip fracture.

**Figure 6:**
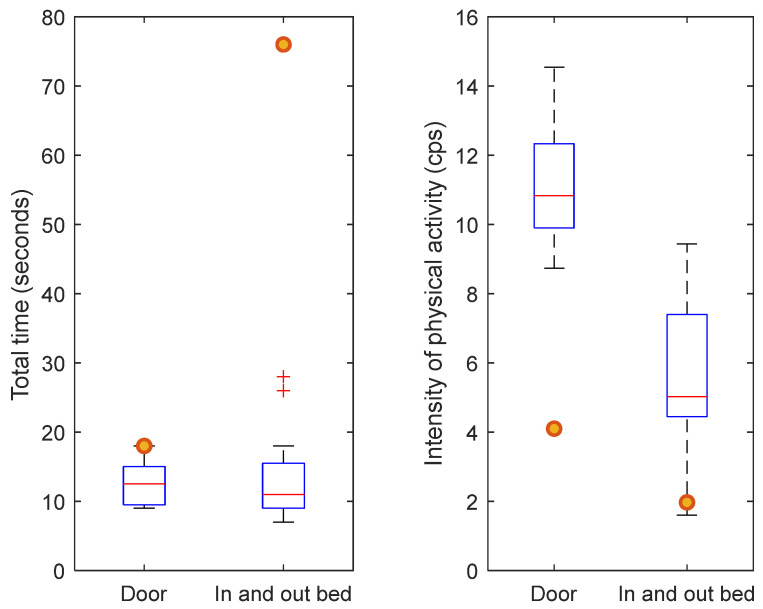
The case participant at the moment of discharge after the hip fracture compared with the total group of participants enrolled in this study. The orange dot represents the case participant. The box plot represents the total group of participants. The left figure shows the results for the total time for walking to the door as well as getting in and out of bed. The right figure shows the results for the intensity of physical activity for both tasks.

The participant who was admitted to our hospital with a hip fracture after participating in this study was 86 years old and had a sedentary lifestyle prior to the hip fracture. The moment of discharge from rehabilitation was exactly two months after hip fracture surgery. The distance to the door at the rehabilitation department was approximately half of the distance to the door at the eHealth House. Despite this, results show a longer total time (18 s) at the moment of discharge compared with the total time (16 s) prior to the hip fracture (Figure 4). The intensity of physical activity was lower at the moment of discharge (4.1 cps at the moment of discharge versus 9.5 cps prior to the hip fracture, Figure 5). For getting in and out of bed, results show a longer total time (76 s) and a lower intensity of physical activity (2 cps) at the moment of discharge compared with the total time (17 s) and intensity of physical activity (3.1 cps) prior to the hip fracture (Figure 4 and Figure 5). Figure 6 shows that both the total time and intensity of physical activity for the single participant at the moment of discharge after hip fracture surgery fall outside the IQR of the community-dwelling older participants.

## 4. Discussion

In this study, mobility and ADL tasks important in the recovery of older patients after hip fracture surgery were quantified with physical activity parameters and measured in 24 community-dwelling older adults. Moving around the house, toileting, getting in and out of bed, and preparing meals was quantified with the following physical activity parameters: total time, time spent sitting, time spent standing, time spent walking, number of transfers, and the intensity of physical activity measured at the upper thigh, lower back, and right foot. A first insight was obtained into the levels of physical activity that are needed in a healthy situation to perform mobility and ADL tasks. Results show the longest total time, time spent standing, and time spent walking for preparing a meal. Moving around the house and getting in and out of bed took the least amount of time but had the highest intensity of physical activity when measured at the thigh and right foot. Furthermore, all tasks were performed without a period of sitting or rest in between, except for getting in and out of bed, which often showed a period of sitting in between the transfer from standing to lying or vice versa. Despite these first insights into the physical activity levels, results also showed a high variability in physical activity parameters between the enrolled participants. Participants with a very active daily lifestyle were able to perform all tasks faster and easier than participants with a sedentary to moderate active daily lifestyle.

By measuring physical activity parameters during mobility and ADL tasks in community-dwelling older adults, a first insight was obtained into the physical activity levels in a healthy situation during tasks relevant to the recovery process after hip fracture surgery. This information can subsequently be used to assess the level of recovery in older hip fracture patients. Results from this study showed high variability in physical activity parameters among the enrolled participants, which was especially seen in the tasks that took more time to perform and for getting in and out of bed. Furthermore, participants with a very active lifestyle were able to perform tasks faster and easier than participants with a sedentary to moderately active lifestyle. This high variability was expected to be found due to the differences between the enrolled participants. It is believed that it also represents today’s society in which there is a high heterogeneity among community-dwelling older adults. From the literature, it is known that the overall heterogeneity in a population increases with age [49]. Therefore, it is suggested that it is more suitable to describe the physical activity parameters necessary to perform each mobility and ADL task by a range of values instead of one average or median value. This range takes into account the differences and variability among older adults. A possible method to describe the range could be the IQR, as the IQR describes the middle 50% of the values and excludes outliers. However, it cannot be concluded yet whether the IQR of each physical activity parameter found in this study is realistic to use for the assessment of the level of recovery. The enrolled participants were generally still very fit as they were able to visit the university by themselves. This can result in a range reflecting higher levels of physical activity parameters, which may not be representative of the community-dwelling older population. The more vulnerable older adults did not participate, which might have influenced the physical activity parameters found in this study. For future research, it is recommended to include a broader spectrum of participants, including those who are less fit and use walking aids. While this will probably increase the heterogeneity of the included participants and broaden the range of physical activity values, it may also influence the generalizability of the study results. A wide range of physical activity values may not present specific subgroups and may limit the applicability of the findings. Therefore, it is recommended to not only enroll a broader spectrum of participants, but also to further assess the factors causing the heterogeneity in the physical activity parameters and to take those factors into account for further analysis. This is also commonly seen in other literature studies, where it was shown that age, comorbidities, and physical functioning can influence physical activity in an older population [6,10,11,12]. Based on the results of this study, the daily physical activity level could be one of those factors affecting the physical activity parameters, and it could be considered to make groups based on those activity levels. Assessing the mobility and ADL tasks for each group separately can support the development of more personalized care strategies.

Results from our case participant showed the comparison between the physical activity parameters measured two months after hip fracture surgery and the physical activity parameters measured in a normal, healthy situation before hip fracture. The total time for walking to the door and getting in and out of bed was higher two months after hip fracture surgery compared with the participant’s total times prior to the hip fracture. Furthermore, the intensity of physical activity was lower for both tasks compared to prefracture measurements. Compared with all enrolled participants, it is also shown that the case participant had a longer total time and a lower intensity of physical activity. This indicates that the participant has not yet recovered two months after hip fracture surgery, even though the participant is able to perform the tasks. Baseline characteristics also showed that the case participant was the least fit participant regarding the daily physical activity levels, which could also explain the results found. Due to the high heterogeneity among community-dwelling older adults, as well as among hip fracture patients, it is considered difficult to compare the results. For this reason, it is also considered important to focus more on the heterogeneity among older adults and to assess the factors causing heterogeneity.

In the past, multiple studies have monitored the recovery of physical functioning in older patients after hip fracture surgery using clinimetric tests [30,50,51,52]. However, continuous monitoring is considered more desirable since clinimetric tests have shown some disadvantages in monitoring a patient’s recovery. Monitoring of the physical function using wearable devices is considered beneficial for both patients as well as healthcare professionals for multiple reasons. First of all, using wearable devices enables objective patient measurements, which are not susceptible to interobserver bias. Second, using wearable devices allows for continuous 24/7 monitoring of the progress and recovery of older adults. In this way, it can be assessed whether a patient is on track or not at multiple moments during the day, and therefore, it is not susceptible to a moment in time. This was also performed for the case participant, who was measured continuously during the whole rehabilitation period at the geriatric rehabilitation department. Last, monitoring physical function by wearable devices is less time-consuming than assessing physical functioning by clinimetric tests. This has a major advantage over the current methods of monitoring physical functioning since the number of older hip fracture patients is expected to increase in the upcoming years due to the aging population, while the number of healthcare professionals is decreasing. To overcome the increasing demand for healthcare and decreasing capacity, wearable devices could be a suitable solution to support the monitoring of physical functioning and to decrease the workload on healthcare professionals. In this current study, the focus was on quantifying physical functioning with physical activity parameters and measuring those parameters in a simulated setting to obtain insight into the level of physical activity in community-dwelling older adults. Most parameters measured in this study were calculated based on the video recordings. However, the ultimate goal is to measure those with wearable devices and to assess physical functioning and the level of recovery in hip fracture patients. Therefore, for future work, it is recommended to emphasize the use of wearable devices containing validated physical activity classification algorithms and have a minimal burden on the older population wearing those devices. Furthermore, it is recommended to develop decision-making tools based on artificial intelligence, which can further support continuous measurements with wearable devices, and to develop feedback systems based on the continuous measurements that provide both patients as well as healthcare professionals more insight into the recovery and that can also assess whether the patient is on track or not.

According to the World Health Organization, physical activity can improve the condition of the muscles and the cardiopulmonary system, increase the functional health status, and reduce the risk of cardiopulmonary diseases, diabetes, cancer, and depression [53]. A decrease in physical activity or sedentary behavior in older adults can result in decreased muscle strength and power [54], a reduced physical function [55], and frailty [56], which all increase the chance of falling and, subsequently, the chance of a hip fracture [57]. Hence, engaging in physical activity is considered important in community-dwelling older adults to reduce the risk of a hip fracture. In this study, it was particularly notable that the participant with a sedentary lifestyle was the one admitted to the hospital with a hip fracture. Possibly, the hip fracture was a result of this sedentary behavior. However, this cannot be concluded based on one participant. Therefore, it may be interesting to further explore the effect of sedentary behavior on the risk of being admitted with a hip fracture. This can be performed by retrospectively assessing the prefracture daily physical activity levels in older hip fracture patients admitted to the hospital and exploring whether there is an association between those levels and the admission to the hospital with a hip fracture or the cause of the hip fracture. Results can subsequently provide valuable insight that can potentially support the development of preventive measures for the community-dwelling older population.

This study also had some limitations. The first limitation was the type of participants enrolled in this study. Despite the fact that we deliberately chose to enroll healthy older adults aged 80 years or older, all enrolled participants walked without a walking aid, were very vital, and, in general, were also very active. This might cause a bias in our study results since it is unsure whether this group of participants is representative of the complete hip fracture population. From previous studies and literature, it is known that there is a significant amount of hip fracture patients who walked with a walking aid prior to the hip fracture and who were not so active in daily life [3,50,58]. Therefore, for future studies it is recommended to also include other types of participants, such as participants with a more sedentary lifestyle or participants using walking aids. Furthermore, one can consider performing the measurements at a different location than the university to increase the chance of enrolling such participants, for example, at the hospital or a local community building, which are often more familiar locations that are more easily accessible.

A second limitation of this study was this study’s study procedure. According to the study procedure, participants were allowed to perform all mobility and ADL tasks in their own order and at their own pace. In this way, we tried to imitate the free-living situation as much as possible. However, some participants wanted to perform all tasks very quickly, which resulted in (1) performing two tasks at the same time or (2) finishing one task and immediately continuing to the next task without sitting in between. This resulted in incomplete tasks, leading to incomplete data for some participants. For future research, more attention should be paid to the correct execution of the study procedure.

## 5. Conclusions

This study measured physical functioning by physical activity parameters in a community-dwelling older population. Mobility and ADL tasks relevant to the recovery process of older hip fracture patients were quantified and measured in order to provide an understanding of those parameters in a normal, healthy situation. Moving around the house, toileting, getting in and out of bed, and preparing meals was quantified with the following physical activity parameters: total time, time spent sitting, time spent standing, time spent walking, number of transfers, and the intensity of physical activity at the upper thigh, lower back, and right foot. The findings of this study gave a first understanding of the levels of physical activity needed in a healthy situation to perform the mobility and ADL tasks and a first comparison with a hip fracture patient two months after hip fracture surgery. This study represents the beginning of more efficient and continuous monitoring of physical functioning, with the ultimate goal of improving the monitoring of physical functioning in older hip fracture patients.

## Figures and Tables

**Table 1:** Description of the mobility and ADL tasks relevant in the recovery of older hip fracture patients.

Needed activity for toileting: 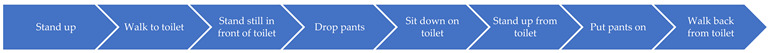
Needed activity for moving around the house: 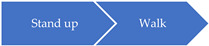
Needed activity for getting in and out of bed: 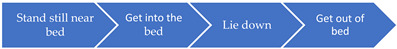
Needed activity for preparing a meal: 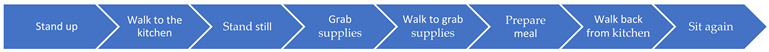

**Table 2:** More detailed information about the physical activity parameters measured during each mobility and ADL task.

		Walk to the Front Door ^a^	Walk to Kitchen ^b^	Walk to Bedroom ^c^	Prepare a Small Meal ^d^	Grab a Drink ^e^	Toileting ^f^	Getting in an Out Bed
Total time (seconds)	Median	13	13	9	186	98	118	11
	IQR	10–15	11–15	8–11	141–201	72–151	77–144	9–16
	Min	9	9	7	94	26	37	7
	Max	18	35	19	326	324	229	28
Time spent sitting (seconds)	Median	0	0	0	0	0	N.A.	0
	IQR	0-0	0-0	0-0	0-0	0-0	N.A.	0–4
	Min	0	0	0	0	0	N.A.	0
	Max	0	0	0	0	0	N.A.	14
Time spent standing (seconds)	Median	0	0	0	132	40	N.A.	0
	IQR	0-0	0–2	0-0	93–153	31–82	N.A.	0-0
	Min	0	0	0	47	5	N.A.	0
	Max	2	20	6	262	220	N.A.	0
Time spent walking (seconds)	Median	10	10	7	44	34	N.A.	0
	IQR	8–12	8–10	6–8	34–54	27–48	N.A.	0-0
	Min	7	6	5	23	15	N.A.	0
	Max	14	13	9	64	114	N.A.	0
Number of transfers (n)	Median	1	1	1	2	2	N.A.	3
	IQR	1-1	1-1	1-1	2-2	2-2	N.A.	2-3
	Min	1	1	1	2	2	N.A.	2
	Max	1	1	1	2	2	N.A.	4
Mean intensity of physical activity thigh (cps)	Median	11	10	8	3	5	3	5
	IQR	10–12	9–11	8–11	3–4	4–6	3–4	4–7
	Min	9	3	6	2	2	2	2
	Max	15	13	15	6	10	6	9
Mean intensity of physical activity lower back (cps)	Median	376	362	350	136	191	171	401
	IQR	319–420	314–386	333–391	115–151	162–239	136–203	321–444
	Min	276	144	265	72	85	96	156
	Max	508	489	546	192	332	267	679
Mean intensity of physical activity on the right foot (cps)	Median	1110	1013	781	274	424	256	637
	IQR	1011–1237	917–1191	656–955	243–358	404–582	192–329	542–821
	Min	854	259	537	112	182	125	143
	Max	1559	1584	1510	495	1070	596	1130

^a^ number of missing = 4, ^b^ number of missing = 2, ^c^ number of missing = 2, ^d^ number of missing = 5, ^e^ number of missing = 5, ^f^ number of missing = 4, N.A. = not applicable.

**Table 3:** Median physical activity parameters are based on the daily physical activity levels.

		Walk to the Front Door ^a^	Walk to Kitchen ^b^	Walk to Bedroom ^c^	Prepare a Small Meal ^d^	Grab A Drink ^e^	Toileting ^f^	Getting in an Out Bed
Total time; median (seconds)	Group 1	9	10	9	153	88	91	9
	Group 2	14	13	10	194	98	120	13
Number of transfers; median (n)	Group 1	1	1	1	2	2	N.A.	2
	Group 2	1	1	1	2	2	N.A.	3

Group 1 = very active lifestyle (n = 8), Group 2 = sedentary to moderately active lifestyle (n = 16), ^a^ number of missing = 4, ^b^ number of missing = 2, ^c^ number of missing = 2, ^d^ number of missing = 5, ^e^ number of missing = 5, ^f^ number of missing = 4, N.A. = not applicable.

## Data Availability

The data presented in this study are available upon request from the corresponding author.
